# Single-Phase Spinel NiCo_2_O_4_ as Highly Active and Stable Electrocatalysts for Urea Oxidation Reaction in Urea Electrolysis

**DOI:** 10.1021/acsomega.5c06513

**Published:** 2025-09-04

**Authors:** Tongxin Zhou, Lihua Zhang, N. Aaron Deskins, Xiaowei Teng

**Affiliations:** † Department of Chemical Engineering, 8718 Worcester Polytechnic Institute, 100 Institute Road, Worcester, Massachusetts 01609, United States; ‡ Center for Functional Nanomaterials, 8099 Brookhaven National Laboratory,Upton, New York 11973, United States

## Abstract

Exploring and designing a stable and active catalyst for the urea electro-oxidation reaction (UOR, CO­(NH_2_)_2_ + 6OH^–^ → CO_2_ + N_2_ + 5H_2_O + 6e^–^) is crucial for the long-term sustainability of ecological systems and clean energy production. We found that spinel NiCo_2_O_4_ is a stable and active electrocatalyst for UOR at a relatively low anodic potential without triggering the competing oxygen evolution reaction (OER). A urea electrolysis cell (CO­(NH_2_)_2_ + H_2_O → CO_2_ + N_2_ + 2H_2_) utilizing a spinel NiCo_2_O_4_ anode and a commercial Pt cathode was further characterized through galvanostatic polarization tests, demonstrating excellent structural stability at various current densities. Post-mortem analysis of long-term urea electrolysis measurements suggested that NiCo_2_O_4_ electrocatalysts maintained a stable spinel structure. However, redistribution of Ni^3+^ to Ni^2+^ valence on the catalyst surface was observed, in contrast to the intact Co valence, indicating that (i) Ni sites are active toward urea adsorption and sequential electro-oxidation; (ii) while urea oxidation proceeds primarily through the direct electro-oxidation mechanism, chemical reactions between the Ni^3+^ site and urea occur during long-term electrochemical UOR operation. Density functional theory (DFT) simulations were used to calculate the adsorption energies of urea molecules on NiO, Co_3_O_4_, and NiCo_2_O_4_, revealing the importance of regulating the configuration of adsorbed urea molecules on the NiCo_2_O_4_ surface.

## Graphical abstract

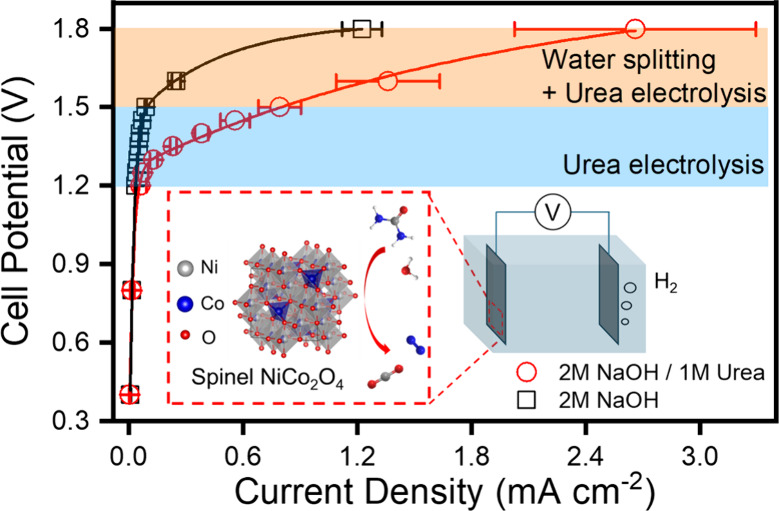

## Introduction

Driven by the depletion of fossil fuels and the need to maintain a healthy environment free from chemical pollution, the development of sustainable energy technologies capable of removing and converting chemical pollutants into electricity or value-added chemicals has gained significant attention.^1,2^ For instance, urea is an energy-enriched compound with high aqueous solubility (1079 g/L at 20 °C), low volatilization, nontoxicity, ideal volumetric energy density (16.9 MJ/L), and high hydrogen content (6.7 wt %).^3^ The improper discharge of urea from various sources, including human activities, industrial manufacturing, and agricultural fertilization, has caused man-made eutrophication in water bodies, where nutrient accumulation leads to a growing population of microorganisms that can deplete oxygen in the water and trigger toxic algal blooms, harming aquatic life and human health.^4^

Urea electrolysis (CO­(NH_2_)_2_ + H_2_O → CO_2_ + N_2_ + 2H_2_) has been considered feasible for treating urea-rich wastewater and producing hydrogen gas, a promising clean fuel for carbon-neutral energy systems.^5^ During electrolysis, the urea molecules are oxidized at the anode via a urea oxidation reaction (UOR, CO­(NH_2_)_2_ + 6OH^–^ → CO_2_ + N_2_ + 5H_2_O + 6e^–^), and the generated electrons transit to the cathode side and react with water to form hydrogen gas via a hydrogen evolution reaction (HER, 6H_2_O + 6e^–^ → 6OH^–^ + 3H_2_). Significant progress has been made in discovering low-cost and effective HER electrocatalysts in alkaline conditions. However, highly active and stable UOR electrocatalysts are considered the bottleneck for implementing urea electrolysis technology. Although the UOR has a lower electrode potential (−0.46 V vs Standard Hydrogen Electrode at pH = 14) than the oxygen evolution reaction (OER, 0.40 V), it suffers from intrinsically high overpotential and sluggish electro-kinetics due to its complex six-electron transfer process. Consequently, a large UOR overpotential makes OER a significant competing reaction when UOR is performed, especially at high current densities.^6^

Great efforts have been made on various Ni-based nonprecious catalysts, including Ni–Co–O, Ni–Mo–O, Ni–Co–Zn-O, Ni_2_Fe­(CN)_6_, Ni–Co–Mn-S, and NiClOH.^7–11^ These catalysts, which have various Ni valence states, show competitive UOR activities compared to precious metal catalysts such as Ni–Ru, Ni–Rh, or Pd.^12^ However, the “true” active Ni valence (e.g., 2+, 3+, or 4+) for UOR remains unclear. Conventionally, it is believed that Ni^3+^ is the active valence state for UOR. However, recent work on Ni_2_Fe­(CN)_6_ indicates low-valence transition metals (e.g., Fe^2+^ and Ni^2+^) are UOR-active.^7^ Conversely, studies on lattice-oxygen-involved UOR suggest Ni^4+^ might be a more UOR-active.^8^ In addition to redox-active species, the UOR mechanism is also subject to debate. It is generally accepted that UOR follows a direct or indirect pathway: the former involves a direct electro-oxidation process on the NiOOH surface,^13^ and the latter involves a chemical reaction between urea and NiOOH to generate Ni­(OH)_2_.^14^ Furthermore, most reported Ni-based catalysts showed mixed metal oxide or hydroxide phases. Therefore, it is still challenging to understand the “real” crystalline phase or Ni-valence states that are pivotal in deciding UOR activity and selectivity against the OER.

Ni and Co can form a stable inverse spinel structure (NiCo_2_O_4_), where Co^2+^/Co^3+^ ions occupy tetrahedral sites and Ni^2+^/Ni^3+^/Co^3+^ ions occupy octahedral sites. Besides offering Ni^2+^/Ni^3+^ and Co^2+^/Co^3+^ redox, NiCo_2_O_4_ also shows higher conductivity than other Co- or Ni-containing minerals because it is generally described as ferrimagnetic and metallic. Thus, it has been intensively used in various electrochemical applications, including energy storage,^15–18^ and sensors.^19,20^ Spinel NiCo_2_O_4_ has also been used as an electrocatalyst for ORR,^21^ OER,^22,23^ formaldehyde, methanol, and ethylene glycol.^24,25^ Given its stable crystalline structure, redox-active Co and Ni species, and, more importantly, the well-defined Ni^2+/3+^ and Co^2+/3+^ distribution within the material, the spinel NiCo_2_O_4_ could be a great model catalyst for understanding the reaction mechanism of UOR. Several studies reported the enhanced performance of UOR on NiCo_2_O_4_ catalysts.^26–31^ However, determining the actual UOR active valence states is still far from settled, especially under urea electrolysis conditions.

Hence, we report a highly stable and UOR-selective NiCo_2_O_4_ spinel catalyst as the anode for urea electrolysis. Benefiting from the synergistic effect between Ni and Co species, our NiCo_2_O_4_ spinel catalyst showed a comparable UOR onset potential (∼0.40 V vs Hg/HgO at the current density of 0.25A m^–2^) compared to the benchmark commercial Pd catalyst (∼0.41 V) and high UOR-selectivity against OER. Besides half-cell characterization, we also studied urea electrolysis in two-electrode full-cell operations using the spinel NiCo_2_O_4_ anode and the commercial Pt cathode. Post-mortem analysis of spinel NiCo_2_O_4_ catalyst after long-term (10 h) urea electrolysis operation suggested coexisting direct and indirect UOR reaction mechanisms. It was found that NiCo_2_O_4_ presented a stable spinel crystalline structure with unchanged Co valences on the catalyst surface. However, nearly 1/3 of the total Ni^3+^ sites on the catalyst surface were reduced to Ni^2+^ after long-term reaction, suggesting a slow but steady chemical reaction between the electrocatalyst and UOR reaction intermediates that caused the reduction of Ni^3+^.

## Experimental Section

### Materials

The following chemicals were used for the synthesis as purchased: nickel nitrate hexahydrate (Ni­(NO_3_)_2_·6H_2_O, 99%, Acros Organics), cobalt nitrate hexahydrate (Co­(NO_3_)_2_·6H_2_O, 99%, Acros Organics), sodium hydroxide (NaOH, 99.99%, ThermoFisher), urea (CO­(NH_2_)_2_, 99%, ThermoFisher), Vulcan XC-72 (Fuel Cell Store), palladium (20% Pd on Vulcan XC-72, Premetek), and Nafion 117 (5%, Sigma-Aldrich). All of the chemicals were used directly without further purification.

### Catalyst Synthesis

Ni­(NO_3_)_2_·6H_2_O and Co­(NO_3_)_2_·6H_2_O with different molar ratios (0.5:0.5, 0.5:1, 1:0.5, 1:0, and 0:1, mmol) were added to a solution containing 75 mL of deionized water, and 5 mmol of urea was then added to the above solution with stirring. The solution was transferred to a Teflon-lined stainless-steel autoclave for hydrothermal treatment at 120 °C for 6 h and then cooled to room temperature under ambient conditions. The product was filtered and washed with deionized water and ethanol, respectively. Afterward, the sample was dried in a vacuum at room temperature for 12 h. Finally, the samples were heated to 400 °C for 3 h in the air to obtain the black powders. The final materials were obtained and named Ni_1_/Co_1_/O (Ni:Co 0.5:0.5), NiCo_2_O_4_ (Ni:Co 0.5:1), Ni_2_/Co_1_/O (Ni:Co 1:0.5), NiO (Ni:Co = 1:0), and Co_3_O_4_(Ni:Co = 0:1).

### Electrochemical Tests

Half-cell measurements were conducted in a CH Instruments 660D/E electrochemical potentiostat using three-electrode half-cells, including glassy carbon drop-coated with active material as the working electrode, platinum wire as the counter electrode, and mercury/mercury oxide as the reference electrode (all from Pine Research Instrumentation). The coating ink was prepared using a 7:3 ratio of active material to XC 72 active carbon mixture in deionized water; 20 μg of active material and 20 μL of 1% Nafion 117 were loaded on the working electrode. The electrolyte consisted of sodium hydroxide and urea, and all electrolytes were degassed using flowing argon gas for at least 30 min before every measurement. The *iR* correction was conducted automatically before each electrochemical testing.

### Structural Characterizations

X-ray photoelectron spectroscopy (XPS) was carried out in a Kratos Axis Supra XPS instrument at the University Instrumentation Center (UIC), University of New Hampshire, using the Al Kα monochromator. The XPS samples weighed 6 mg with a 7:3 ratio of active material and carbon black loaded on the carbon papers and were collected after conducting three-electrode measurements. These electrodes were stopped at different potentials studied in an ultrahigh vacuum of approximately 10^–8^ Torr. CasaXPS software was used to process and analyze the obtained results, and all of the spectra were calibrated according to the adventitious carbon (C 1s) peak at a binding energy of 284.8 eV.

Scanning electron microscopy (SEM) was performed with FEI Quanta 200 FEG MKII at UMass Chan Medical School.

Scanning transmission electron microscopy (STEM) was conducted at the Electron Microscopy Facility at the Center for Functional Nanomaterials in Brookhaven National Laboratory. The instrument used for the high-angle annular dark-field (HAADF) image was the FEI Talos F200x scanning/transmission electron microscope equipped with an X-FEG electron source module and operated at 200 keV. The elemental mapping of the discharge sample was done by a four-quadrant 0.9 sr energy-dispersive X-ray spectrometer (EDS).

Synchrotron X-ray diffraction (XRD) studies were conducted at beamline 28-ID-1 of the Brookhaven National Laboratory. The XRD images were collected on a 2D array detector. All of the acquired patterns were phase analyzed by the Rietveld refinement using GSAS-II software. The synchrotron instrument parameters were calibrated by the peak fitting of the CeO_2_ standard. The radiation wavelength is 0.1665 Å.

X-ray absorption spectroscopy (XAS) measurements were done at beamline 6-BM for Materials Measurement at the National Synchrotron Light Source-II, Brookhaven National Laboratory. The XAS measurements were carried out in transmission mode at the Ni and Co K-edge. Metal foil and metal oxide powders were used as references for X-ray energy calibration and data alignment. Athena software from the Demeter package was used to perform XAS data processing and analysis.

### Density Functional Theory Simulations

We used the Vienna ab initio Simulation Package (VASP) for all DFT simulations.^32–35^ Core electrons were represented by Projector Augmented-Wave (PAW) potentials.^35,36^ Valence electrons were modeled using a plane wave basis set with a cutoff energy of 450 eV. We utilized the PBE functional^37^ in combination with the + U method.^38^ The + U method is often helpful for better describing metal oxides. We applied the following U values in the three materials: 4.4 eV (tetrahedral Co), 6.7 eV (octahedral Co), and 6.6 eV (Ni), similar to literature values.^39,40^ Gaussian smearing with a sigma value of 0.05 eV was used. Dispersion forces were accounted for through the Grimme D3 correction.^41^

All calculations were performed with (100) surface slabs, being at least 8 Å in length along the *x* and *y* directions, and sufficient vacuum space in the z direction (>10 Å). We used a Monkhorst–Pack 4 × 4x1 k-point mesh for all the surface calculations.^42^

There may be several possible magnetic states for each metal oxide, and different magnetic states lie on different energy contours. Literature was used as a guide to select initial magnetic moments and surface terminations.^39,43–45^ To ensure consistency in the magnetic states, we modeled urea adsorbed onto the surface and then slowly raised the urea away from the surface in a controlled stepwise process until the urea was a sufficient distance from the surface and in the vacuum region between slabs. The WAVECAR from the previous step was used for each subsequent step. This process helped ensure that the initial configuration (urea adsorbed on the surface) and the final configuration (urea desorbed from the surface) have similar electronic and magnetic states. The adsorption energy of urea is simply the energy difference between the initial and final energies.

## Results and Discussion

### Material Synthesis and Structure Characterizations

A solvothermal method was used to produce various Ni and Co mixed oxide compounds in urea and water solution at 120 °C in an autoclave, followed by thermal treatment at 400 °C in air. Figure 1a shows the crystalline structures of Ni/Co oxides with Ni: Co precursor molar ratios were 1:2, 1:1, and 2:1. Synchrotron X-ray diffraction (XRD) analysis confirms that spinel NiCo_2_O_4_ formed when a Ni/Co precursor molar ratio of 1:2 was used. Under other precursor ratios, NiCo_2_O_4_ and Co_3_O_4_ spinel, NiO, and CoO mixtures were observed. The ratio of NiCo_2_O_4_/Co_3_O_4_ to CoO/NiO is calculated by Rietveld refinement using the NiO and NiCo_2_O_4_ standard phases. Notably, NiO and Co_3_O_4_ formed under the same synthetic conditions when Co and Ni precursor ratios were 0:1 and 1:0 (Figure S1).

**Figure 1:**
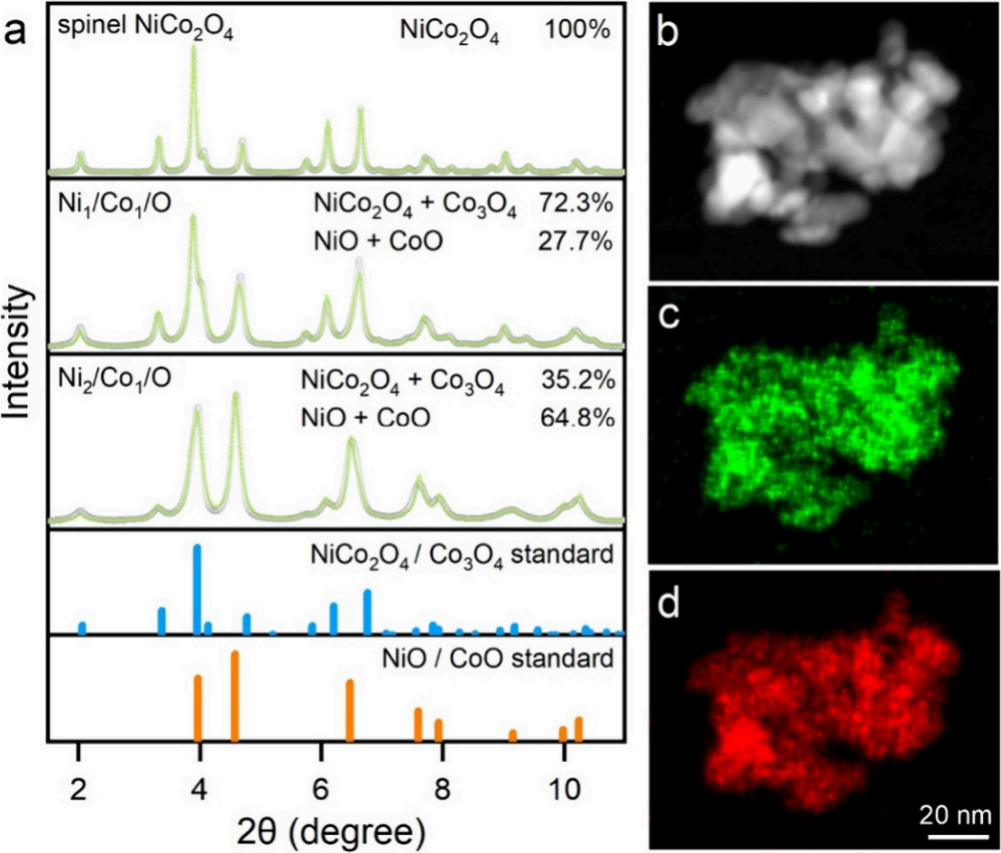
(a) XRD patterns of Ni/Co oxides with different Ni and Co ratios and (b) HAADF image of NiCo_2_O_4_ with the element mapping of (c) Ni and (d) Co.

Notably, XRD alone cannot confirm the formation of pure-phase NiCo_2_O_4_ spinel from Co_3_O_4_ spinel, because both share very similar diffraction patterns. However, Ni and Co have distinct energy-dispersive X-ray spectroscopy (EDS) peaks, which can be resolved with modern detectors equipped in scanning transmission electron microscopy (STEM). Figure S2 shows a STEM image of large-area particle assemblies, where the NiCo_2_O_4_ particles exhibit an average size of ∼10 nm. Figure 1b–d shows the high-angle annular dark-field (HAADF) image and the elemental distribution of Ni and Co within individual NiCo_2_O_4_ particles, characterized by the STEM equipped with EDS. The EDS elemental mapping signals from Ni and Co reveal a homogeneous Ni and Co distribution within the individual particles with a Ni/Co molar ratio of 1:2, consistent with the XRD analysis and precursor ratio employed during the synthesis. Figure S3a is a SEM image of a large-area NiCo_2_O_4_ assembly, showing “urchin-like” morphology, in accordance with the reported morphology of spinel NiCo2O4 made by hydrothermal methods.^27,46^

### UOR Performance in Half Cells

The electrocatalytic properties of spinel NiCo_2_O_4_ were tested compared with benchmark commercial Pd/C catalysts. All the current density data of each catalyst presented in this study are averaged by their electrochemically active surface areas (ECSAs) values by measuring the double-layer capacitance of the catalytic surface. ECSA measurements were conducted specifically within the nonfaradic current region using cyclic voltammetry in 0.1 M NaOH solution, as shown in Figure S4 and Table S1. The electrocatalytic performance of the Ni/Co spinel was first tested in 2 M NaOH with and without 1 M urea using linear sweep voltammetry (LSV).

Figure 2a shows the LSV of spinel NiCo_2_O_4_ in a 2 M NaOH solution. The redox features are exclusively attributed to OER, where the OER current increases exponentially when the potential exceeds ∼0.6 V. In contrast, an additional redox feature appears in the solution containing 2 M NaOH and 1 M urea when the applied potential is beyond 0.4 V, which could be exclusively attributed to UOR. As the potential increases beyond 0.6 V, the anodic current increases rapidly, similar to the control LSV conducted in 2 M NaOH electrolytes. Figure 2b shows cyclic voltammetries (CVs) of all three Ni/Co oxides in 2 M NaOH and 1 M urea with both forward and backward scans. The anodic current in the backward sweep indicates continuous oxidation of intermediate residues on the catalyst’s surface generated during the prior electro-oxidation process in the forward sweep. The spinel NiCo_2_O_4_ shows the lowest anodic current in the backward sweep, suggesting a more complete UOR and less reaction intermediate build-up in the previous forward sweep than other Ni/Co oxides containing spinel NiCo_2_O_4_, Co_3_O_4_, NiO, and CoO mixtures. Notably, the Pd/C catalysts show the least anodic current in the backward sweep in comparison to three Ni/Co oxides (including spinel NiCo_2_O_4_), suggesting a more complete UOR reaction in the anodic sweep. However, spinel NiCo_2_O_4_ shows superior UOR electro-kinetics than other Ni/Co oxides by its lower onset potential (∼0.40 V at the current density of 0.25 A m^–2^), even comparable to that of commercial Pd/C catalysts (∼0.41 V), as shown in Figure 2c. Moreover, spinel NiCo_2_O_4_ shows a lower Tafel slope (87.8 mV dec^–1^) compared to other electrocatalysts, as shown in Figure 2d.

**Figure 2:**
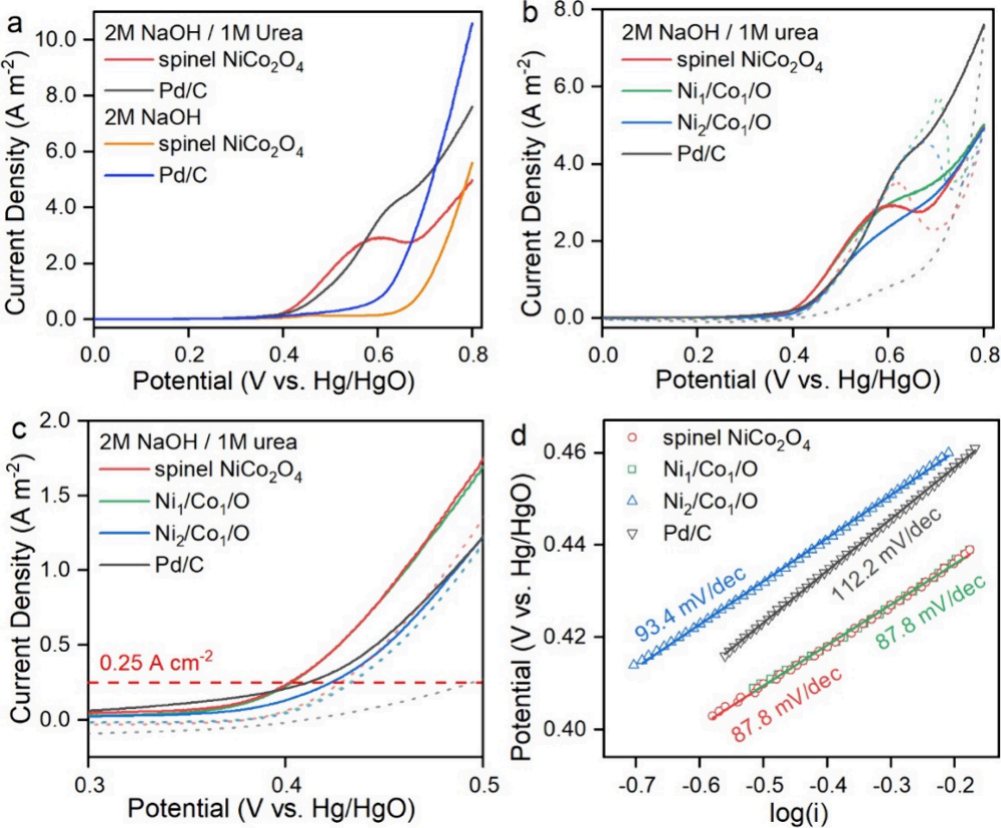
Electrochemical properties of spinel NiCo_2_O_4_, other Ni/Co oxides, and commercial Pd/C catalysts. (a) LSVs in 2 M NaOH/1 M urea and 2 M NaOH. (b) CVs in 2 M NaOH/1 M urea (solid lines: the forward sweep, dashed lines: the backward sweep). (c) Zoom-in LSV to show the potential of various catalysts at a current density of 0.25 Am^–^^2^, and (d) Tafel plots and corresponding Tafel slopes.

To understand the charge transfer kinetics of catalysts with various Ni/Co compositions, electrochemical impedance spectroscopy (EIS) was performed (Figure S5). The charge transfer resistance (Rct) was determined from the Nyquist plot, showing an increasing order of NiCo_2_O_4_ < Ni_1_/Co_1_/O < Ni_2_/Co_1_/O, which is congruent with the trend observed by LSV and Tafel measurements. Additionally, Figure S6 shows the EIS of NiCo_2_O_4_, Ni_1_/Co_1_/O, and Ni_2_/Co_1_/O catalysts after UOR at 0.4 V. The results showed that Rct values of the three catalysts significantly increased after UOR compared to the unreacted ones, suggesting the accumulation of the reaction residues on the catalyst surfaces. However, the NiCo_2_O_4_ catalyst still showed the lowest Rct, suggesting a more complete UOR compared to other catalysts.

Figure 3a shows the staircase voltammetries in the solutions of 2 M NaOH/1 M urea and 2 M NaOH of various catalysts, from which the contributions of UOR and OER to the overall current density at a given potential can be quantified.^47^ In staircase voltammetry, the potential is swept in a series of steps, and the current is measured at the end of each potential step after 200 s. Therefore, all the current densities in Figure 3 are from the electro-oxidation processes (e.g., UOR and OER), excluding the non-Faradaic capacitive current.

**Figure 3:**
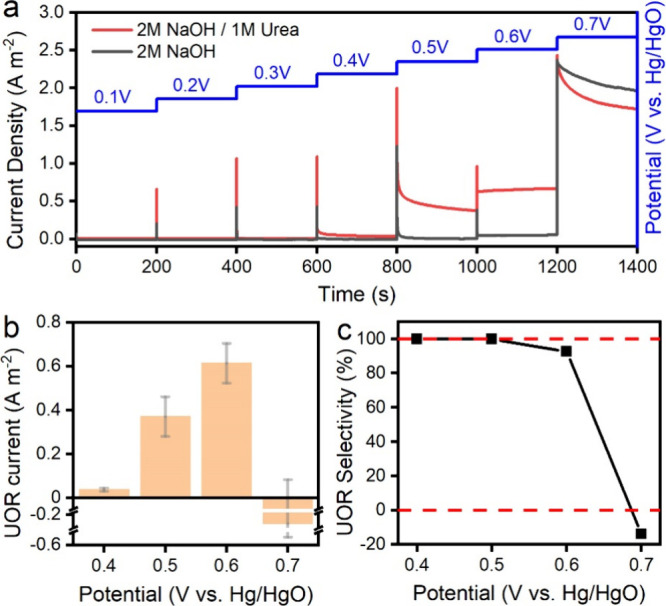
(a) Staircase voltammetry of Ni/Co spinel in 2 M NaOH/1 M urea and 2 M NaOH. (b) UOR current and (c) selectivity of UOR.

We calculated the current and selectivity of UOR from staircase voltammetry (SV) analysis at each potential stair step, as shown in Figure 3b,c. The overall anodic current density (*i*_A_) is obtained from SV in the NaOH/urea electrolyte, the OER current density (*i*_OER_) is obtained from SV in the NaOH electrolyte, and the UOR current density is then calculated (*i*_UOR_ = *i*_A_ – *i*_OER_). The selectivity of UOR (S_UOR_) at each potential is then estimated by the ratio of *i*_UOR_ to *i*_A._ Our results show that the SV current (*i*_A_) can be exclusively attributed to UOR at 0.40 and 0.50 V, where the OER remains inactive in such a low potential range. When the potential increased to 0.60 V, a low OER current but a remarkable UOR current response can be observed. When the potential increases to 0.7 V, the OER current contributes dominantly to the overall current.

Notably, *i*_UOR_ and S_UOR_ are calculated by assuming that UOR and OER occur independently on the catalyst surface. While this assumption is valid within the lower potential range (0–0.6 V), it is not necessarily accurate in higher potential ranges (>0.6 V). Beyond 0.6 V, we observed a competitive interaction of urea and water molecules on the catalyst surface, as evidenced by the fact that *i*_OER_ surpasses the overall current, causing *i*_UOR_ to become negative at 0.7 V. Since a negative current implies a cathodic process, this is not a meaningful representation of the anodic current. Instead, this observation confirms a competitive adsorption mechanism between water and urea molecules on the surface of Ni/Co oxide and hydroxide heterostructure catalyst at high potentials (>0.6 V). Even when the OER dominates at high potentials, residual urea molecules may still occupy active sites, hindering the OER kinetics, evidenced by *i*_OER_ exceeding the overall current. In future studies, gas-phase product analysis will be conducted to provide a more accurate estimation of the S_UOR_.

A comparison of the UOR performance of spinel NiCo_2_O_4_, other Ni/Co oxides, NiO, Co_3_O_4_, and commercial Pd/C catalysts in staircase voltammetry is shown in Figure S7. Spinel NiCo_2_O_4_ showed superior UOR activity and selectivity against the OER to other Ni/Co oxides and commercial Pd/C at a low potential range, especially at 0.50 V.

### Full-Cell Urea Electrolysis Measurements

A two-electrode urea electrolysis cell was constructed and tested by using the spinel NiCo_2_O_4_ anode and the commercial Pt cathode with an electrolyte volume of 5 mL at room temperature under atmospheric pressure (see Figure S8 for the electrolysis cell design). Figure 4a shows the multiple potential steps of the electrolysis cell, ranging from 0.4 to 1.8 V. The current densities are collected at the end of each potential step after a 30 min operation for the resulting polarization curves (*I*–*V* curves), as shown in Figure 4b. The results from the urea electrolysis cell using 2 M NaOH and 1 M urea solution are presented, compared to the water electrolysis cell under the same conditions except for using 2 M NaOH solution. Water electrolysis (black) and urea electrolysis (red) have similar current density vs cell potential trends, where electrolysis current density increases with increasing cell potential. However, the onset cell potential for water electrolysis is around 1.5–0.25 V higher than that of urea electrolysis. Therefore, the urea electrolysis cell can operate exclusively via UOR at the anode at a potential of 1.4 V without interference from OER, yielding a current density of 0.38 mA/cm^2^.

**Figure 4:**
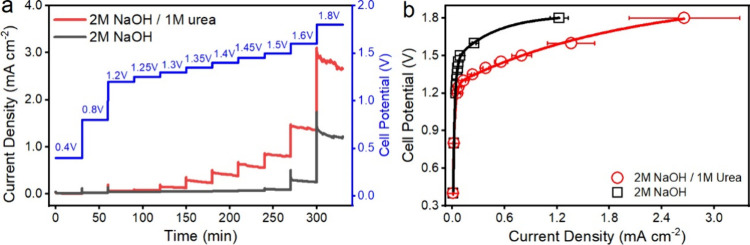
Full-cell urea electrolysis performance: (a) Multiple potential step voltammetries and (b) cell polarization curves in NaOH solution (water electrolysis) and NaOH/urea solution (urea electrolysis).

### Catalyst Stability in Long-Term Operation

The stability of the spinel NiCo_2_O_4_ catalyst was also evaluated by post-mortem structural analysis after 10 h of UOR performance at a constant potential of 0.5 V (vs Hg/HgO) in a half-cell (Figures S9).

First, the synchrotron XRD measurements show that spinel NiCo_2_O_4_ after 10 h of UOR has the same crystalline phase and grain size (∼9.1 nm, calculated using the Scherrer formula) as the pristine catalysts, as shown in Figure 5a. In addition to the bulk crystalline structure, the X-ray absorption near-edge structure (XANES) also suggests that the bulk electronic structure of spinel NiCo_2_O_4_ remains stable. Figure 5b–e shows the K-edge features of Ni and Co in the bulk structure after long-term UOR, compared to pristine materials. Note that the NiCo_2_O_4_ catalysts in the pristine state and after 10-h UOR measurements remain nearly identical Ni and Co K-edge features (Zoom-in XANES spectra are shown in Figure S10), suggesting the bulk electronic structure of the catalysts does not change after long-term reaction. The valence values were calculated from the linear fit of the absorption energy at the half-edge step [12μ(E)] between Ni^2+^ and Ni^3+^ from Ni^II^O and LaNi^III^O_3_ standards and between Co^2+^ and Co^2+/3+^ from Co^II^O and Co^III^_2_Co^II^O_4_ spinel standards (Figure S11). Likewise, Ni and Co components show the average valence of +2.51 and +2.81 after 10 h UOR measurements, respectively, consistent with those at pristine states. Further support for the spinel NiCo_2_O_4_ catalyst can be acquired from the local structure change by Fourier Transform EXAFS in R space from Figure 5d,e. Based on the radial distribution function of the central Ni or Co atom, the distinct peaks at ∼1.5 and ∼2.5 Å can be identified as M–O (M: Co or Ni) and M–M backscattering, respectively. Almost identical curves at all potentials in R-space demonstrated that chemical bonding structure (e.g., M-M and M-O bond length) remained unchanged for both species before and after long-term UOR. Therefore, the spinel NiCo_2_O_4_ catalyst exhibited a highly stable bulk structure and valence states.

**Figure 5:**
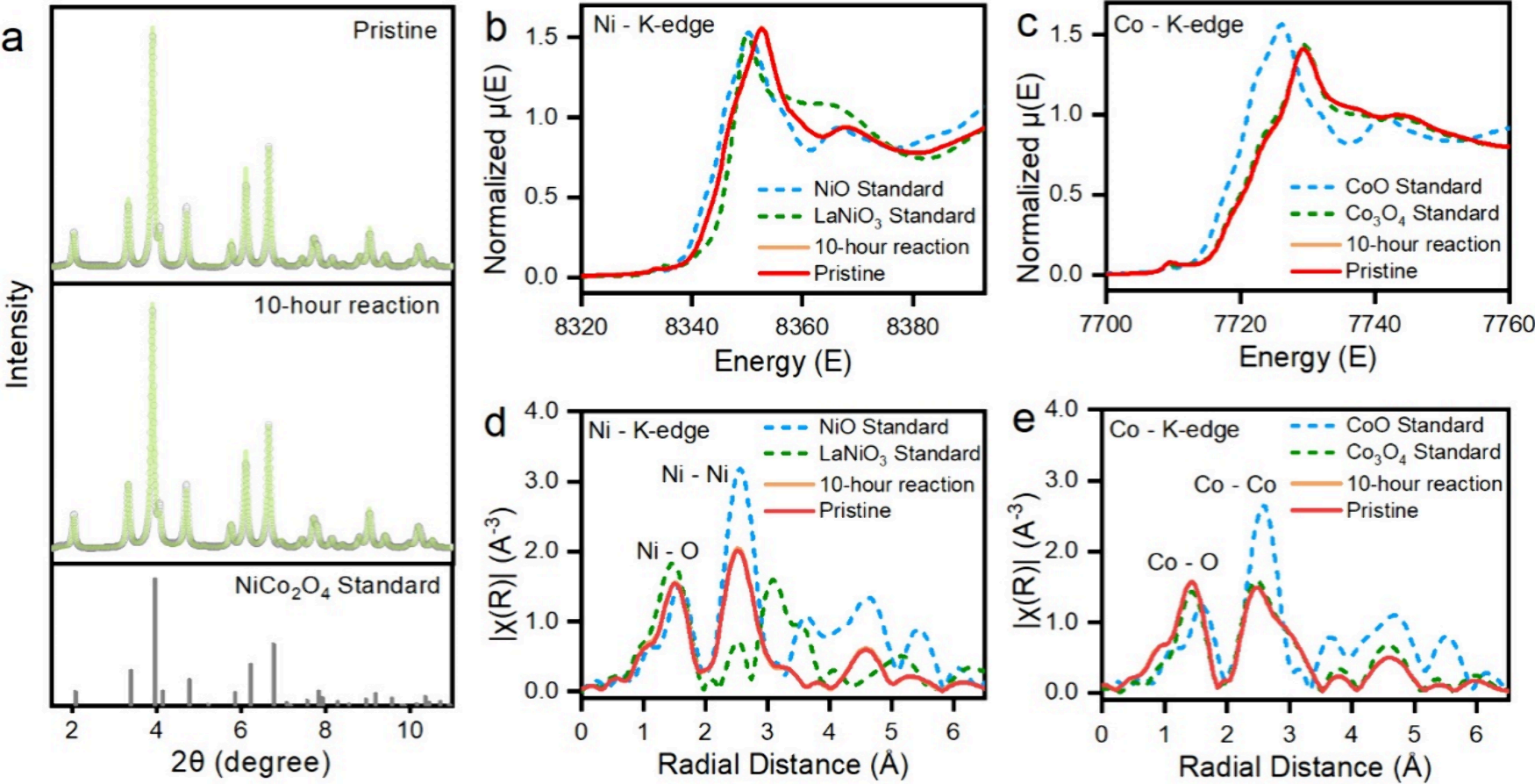
(a) XRD pattern of the spinel NiCo_2_O_4_ catalyst at the pristine state and after 10 h UOR measurements at 0.5 V, and XANES spectra of (b, d) Ni K-edge and (c, e) Co K-edge in energy space and radial space.

Second, the surface valence states of the spinel NiCo_2_O_4_ catalyst after long-term UOR were studied by X-ray photoelectron spectroscopy (XPS), compared to the pristine catalyst. Figure 6 shows the Ni- and Co-2p_3/2_ spectra of spinel NiCo_2_O_4_ before and after the 10 h UOR test. The full spectrum is shown in Figure S12. The Ni 2p_3/2_ spectrum of NiCo_2_O_4_ shows peaks at 854.5 and 856.1 eV, coinciding with the metallic Ni^2+^ and Ni^3+^ components, respectively. The broad peak at ∼861 eV is ascribed to the shake satellite, arising from the ionization of the core ions to excited states. Note that the high peak intensity of this satellite feature is primarily due to strong electron correlation effects in Ni^2+^ species, where the core hole generated during emission of the 2p electron interacts with the 3d electron, leading to multiplet splitting and intensification of the peak. Similarly, Co 2p_3/2_ peaks at 780.2 and 781.8 eV belong to Co^3+^ and Co^2+^ species, as well as the shake satellite peaks at ∼783 and ∼789 eV. The resulting ratios of Ni^3+^/Ni^2+^ and Co^3+^/Co^2+^ were analyzed and summarized in Table 1. The Co^3+^/Co^2+^ ratio remains nearly constant before and after the long-term UOR measurements. In stark contrast, the Ni^3+^/Ni^2+^ ratio decreased from 1.41 to 0.65, suggesting that 32.6% of Ni^3+^ ions on the catalyst’s surface were reduced to Ni^2+^ after the 10 h UOR measurements. However, the catalyst retains highly stable bulk crystalline and electronic properties, as shown by XRD and XANES in Figure 5. As illustrated in Figure 7, these observations suggest that (i) while a significant UOR is carried out via an electrochemical process, the chemical reaction between urea and the catalysts occurs. Likely, Ni^3+^ components on the catalyst’s surface were chemically reduced by reaction intermediates simultaneously generated during the electrochemical oxidation of urea; (ii) the UOR current density sustained after the 10-h UOR operation, accompanied by the surface Ni valence redistribution from 3+ to 2+, strongly infers that Ni^2+^ sites are active toward UOR, consistent with our previous published result;^47^ (iii) Ni sites (Ni^3+^ and Ni^2+^) are responsible for urea absorption and consequential urea oxidation reaction (electrochemically and chemically), while Co sites might primarily interact with water molecules. This bifunctional effect of Ni/Co species in NiCo_2_O_4_ is consistent with previous reports.^28^

**Table 1:** Ratios of Ni^3+^/Ni^2+^, Co^3+^/Co^2+^, and M–OH/M-O for Ni/Co Spinel Catalyst Obtained from XPS

	Ni^3+^/Ni^2+^	Co^3+^/Co^2+^	M–OH/M-O
pristine	1.41	1.96	4.20
long-term holding	0.65	2.01	5.03

**Figure 6:**
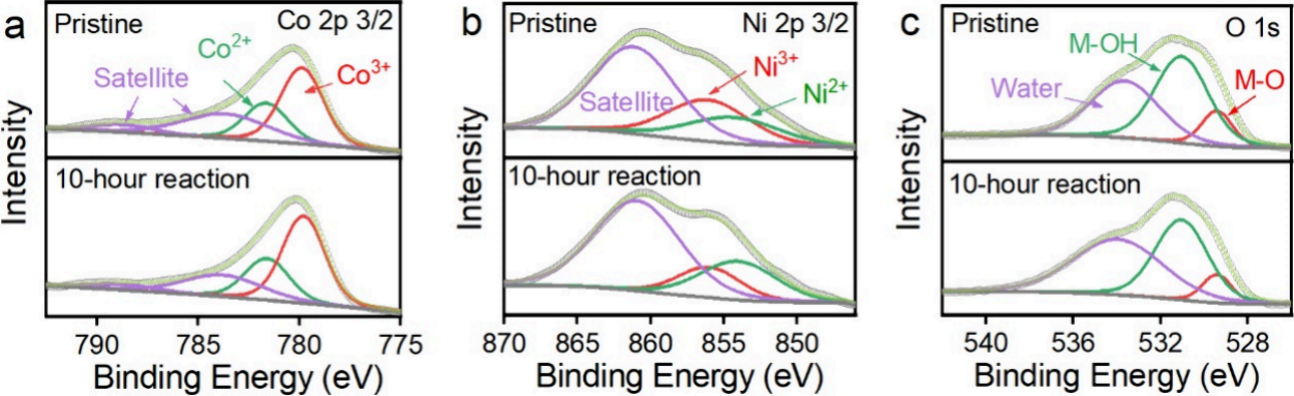
XPS spectra (a) Co-2p_3/2_, (b) Ni-2p_3/2_, and (c) O-1s components in spinel NiCo_2_O_4_ catalyst before and after long-term UOR.

**Figure 7:**
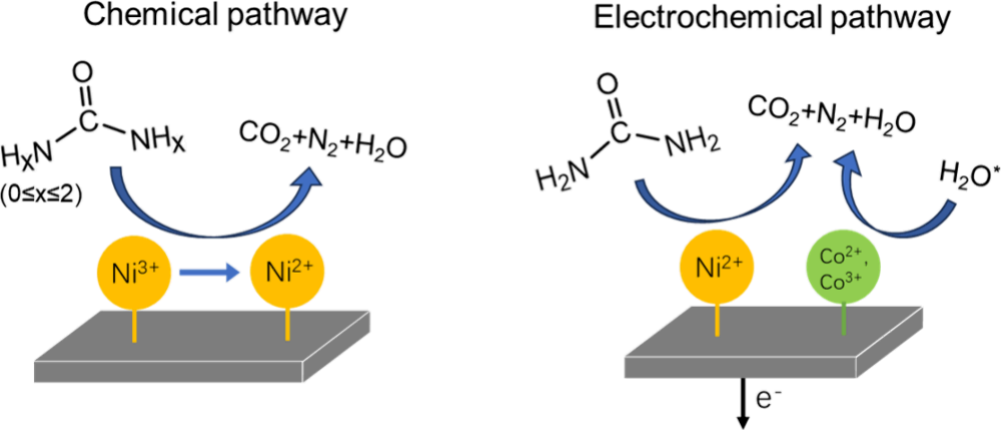
Mechanism diagram of the proposed bifunctional mechanism in UOR (* denotes the absorbed water).

Figure 6c shows the XPS spectra of O 1s of spinel NiCo_2_O_4_ catalyst before and after long-term UOR. There are three major components at ∼533.7, 531.0, and 529.4 eV, respectively.^27,48^ The component at 529.4 eV of the A-1s can be attributed to the typical oxygen fingerprint from metal–oxygen bonds. The component at 531.0 eV is associated with the hydroxyl group. The ratio of metal-hydroxyl and metal–oxygen (M–OH/M-O) increased from 4.20 to 5.03 (Table 1) after the 10-h UOR measurements, consistent with the above XPS Co and Ni results showing the reduction of Ni^3+^ to Ni^2+^ owing to the chemical reaction with urea. Finally, the contribution of the O-1s at 533.7 eV resulted from physically and chemically absorbed water on the surface.

Third, SEM imaging of NiCo_2_O_4_ after 10-h UOR showed similar “urchin-like” morphologies, compared to the pristine catalyst, congruent with the expected high structural stability of spinel materials (Figure S3b). STEM-EDS mapping reveals a homogeneous distribution of Ni and Co within the NiCo_2_O_4_ particles after 10 h of UOR operation (Figure S13), consistent with the pristine state shown in Figure 1. This observation confirms that the NiCo_2_O_4_ phase remains structurally stable during the long-term UOR measurements. However, the Ni: Co molar ratio increases from 0.5 to 0.6 after the reaction, indicating a preferential leaching of cobalt species under UOR conditions compared to Ni counterparts.

### DFT Calculations to Understand NiCo_2_O_4_ for UOR

We identified the dynamic valence changes of Ni and Co sites on the NiCo_2_O_4_ catalyst surface during the UOR by XPS analysis, suggesting that both Ni and Co sites are responsible for the high catalytic activity. We further conducted DFT calculations to depict the adsorption process of urea molecules on various catalyst surface configurations to provide insight into the thermodynamics of potential catalytic reactions. For simplicity, the (1 0 0) surfaces of NiO, Co_3_O_4_, and NiCo_2_O_4_ were selected for calculation because they are the most stable facets.^39,43,49,50^ Calculated by DFT, the most stable coordination of urea molecules on these facets is identified and shown in Figure 8.

**Figure 8:**
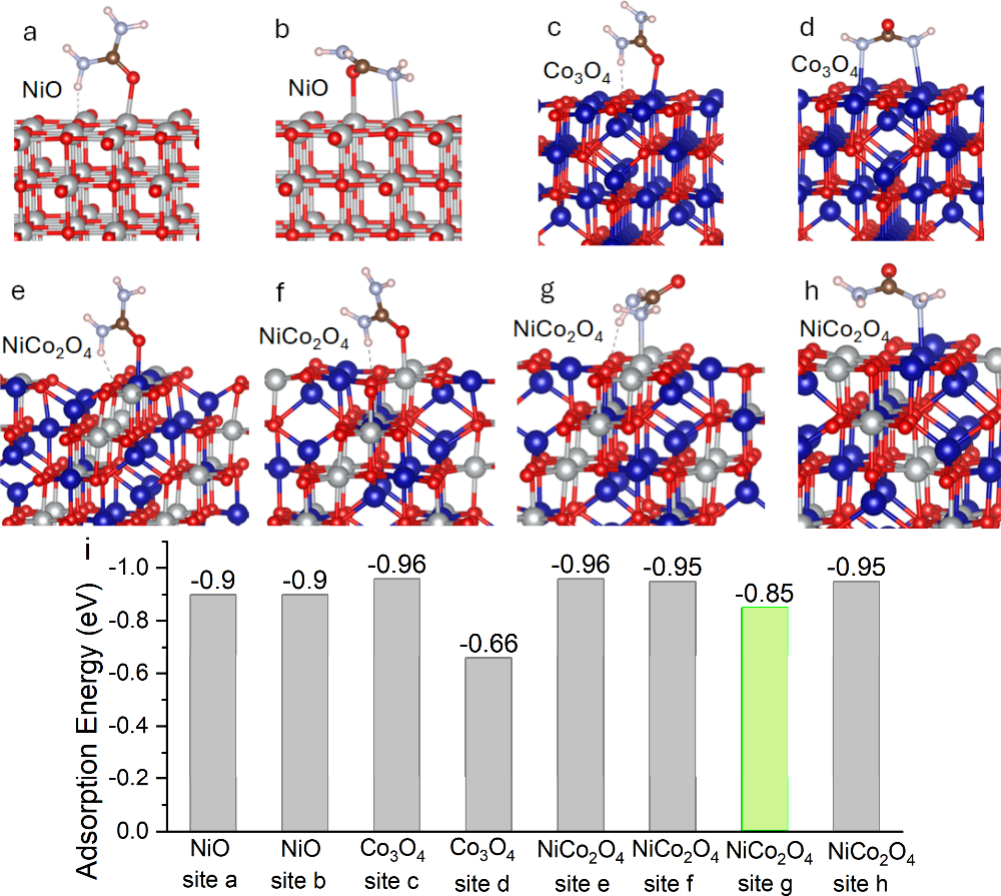
Configurations of urea molecule adsorption on (001) planes of (a, b) NiO, (c, d) Co_3_O_4_, and (e-h) NiCo_2_O_4_, and (i) the calculated adsorption energies of urea molecules on different catalyst surfaces. A reasonably moderate adsorption energy (not too strong or weak) is highlighted in green for site g on NiCo_2_O_4_ (blue: Co; gray, Ni; red: O; brown: N; pink: H; light blue: N).

Our DFT calculations show that Ni and Co atoms interact with either the nitrogen or oxygen atom of the urea molecule, as shown in Figure 8a–h. This is consistent with the literature, revealing nitrogen or oxygen bonding with transition metal ions such as Pd, Pt, Cr, Fe, Zn, and Cu upon the formation of a urea-metal complex.^51^ The calculated adsorption energies in these configurations are compared in Figure 8i. Notably, Co_3_O_4_ has the weakest interaction with urea when two nitrogen atoms of a urea molecule bridge over adjacent Co sites (Figure 8d), showing the highest urea adsorption energy of −0.66 eV. Similarly, Co_3_O_4_ and NiCo_2_O_4_ show the most substantial urea interaction when oxygen atoms interact with Co or Ni sites (Figure 8c,e,f) or nitrogen atoms interact with Co sites (Figure 8h), giving the lowest adsorption energies of −0.95 or −0.96 eV. Notably, Sabatier’s principle points out that the reactants should be moderately adsorbed on active sites for achieving the best performance. At the same time, adsorption that is too strong or too weak is not suitable for catalytic interactions. As a result, the DFT calculations suggest that the interaction between nitrogen atoms and the Ni site on the NiCo_2_O_4_ surface might be the most probable metal-urea configuration for the best UOR, given its moderate urea adsorption energy (−0.85 eV). UOR is a complicated reaction involving six consecutive elementary steps. Interactions between urea and active sites intrinsically regulate the complete oxidation of the urea molecule. Although the simple calculation of adsorption energies reported here may not explicitly explain the underlying mechanism of the superior performance of NiCo_2_O_4_, our studies highlight how the initial configuration of adsorbed urea molecules could strongly affect the thermodynamics and kinetics of the following elementary steps.

## Conclusions

We studied a spinel NiCo_2_O_4_ catalyst with an average size of ∼10 nm as a model catalyst featuring a well-defined Ni (2+/3+) and Co (2+/3+) distribution within the particles for UOR. Electrochemical analysis validates the high activity and selectivity of NiCo_2_O_4_ toward UOR against the OER at a low potential range (≤0.50 V vs Hg/HgO), superior to benchmark commercial Pd/C catalysts. While sustaining UOR activity and maintaining a stable crystalline structure and valence properties in the bulk state after long-term UOR operation, evidenced by synchrotron XRD and XAS analysis, nearly 1/3 of Ni^3+^ sites on the catalyst were reduced to Ni^2+^, while surface Co species remained unchanged. This observation suggests that Ni^2+^ might be an active site for UOR, while the Co sites (Co^2+^ or Co^3+^) have a weak interaction with urea molecules at the surface (Figure 8). DFT calculations on the adsorption energies of urea molecules on NiO, Co_3_O_4_, and NiCo_2_O_4_ further revealed the importance of regulating the configuration of adsorbed urea molecules on the Ni/Co surface. This work revealed the essential structure for the stable catalyst and sheds light on the surface treatment needed to improve the catalytic performance of UOR.

## Supplementary Material



## Notes

SEM images, EIS measurements, STEM-EDS mapping, and additional electrochemical measurements (PDF)
