# Supplementary material for: Single-Phase Spinel NiCo2O4 as Highly Active and Stable Electrocatalysts for Urea Oxidation Reaction in Urea Electrolysis
Source: ACS Omega. 2025 Sep 4;10(36):41917–25. doi: 10.1021/acsomega.5c06513 (PMC12444578; doi:10.1021/acsomega.5c06513)
Supplement: Supplementary file 1 [file ao5c06513_si_001.pdf]

Supporting Information

Single-Phase Spinel $NiCo_2O_4$ as Highly Active and Stable Electrocatalysts for Urea Oxidation Reaction in Urea Electrolysis

Tongxin Zhou, [a] *Lihua Zhang,* [b] *N. Aaron Deskins,* [a] *and Xiaowei Teng** [a]

a Department of Chemical Engineering, Worcester Polytechnic Institute, 100 Institute

Road, Worcester, MA 01609, United States

b Center for Functional Nanomaterials, Brookhaven National Laboratory, Upton, New York 11973, United States

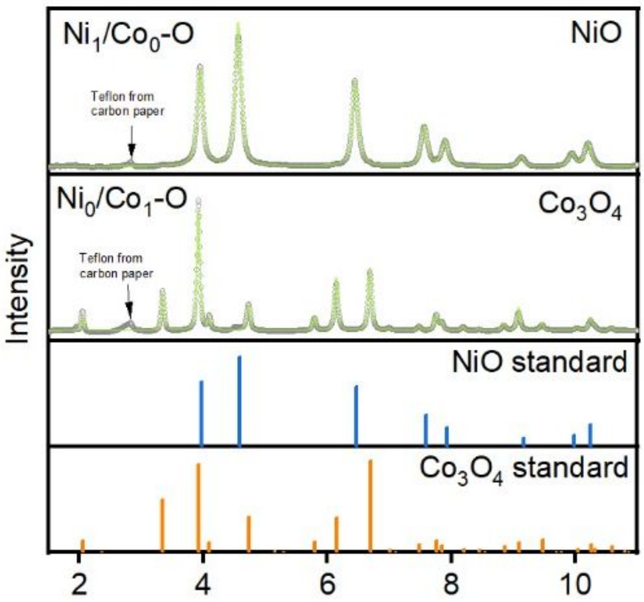

Figure S1. XRD patterns of Ni_1/Co_0 and Ni_0/Co_1 samples, showing pure-phase NiO and Co_3O_4 crystalline materials.

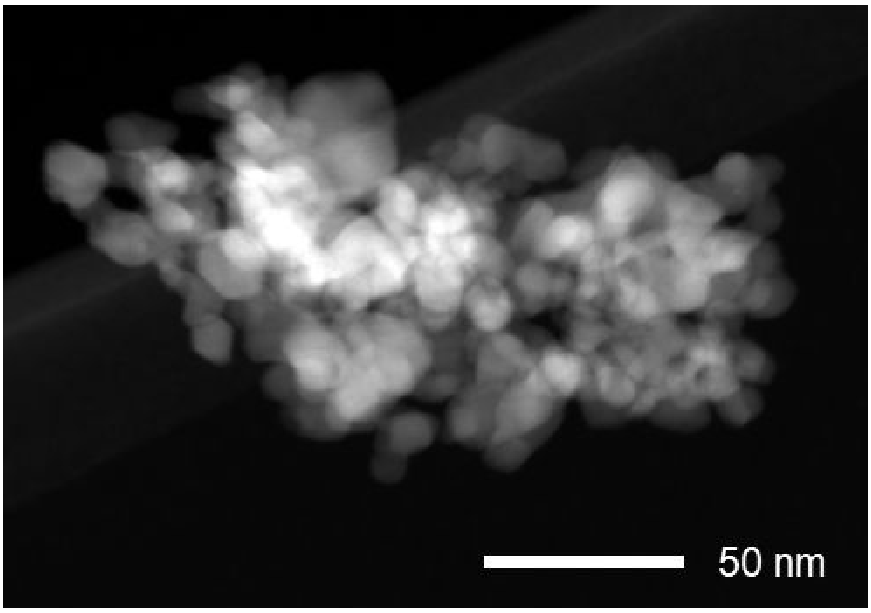

Figure S2. STEM image of NiCo2O4 oxide particles.

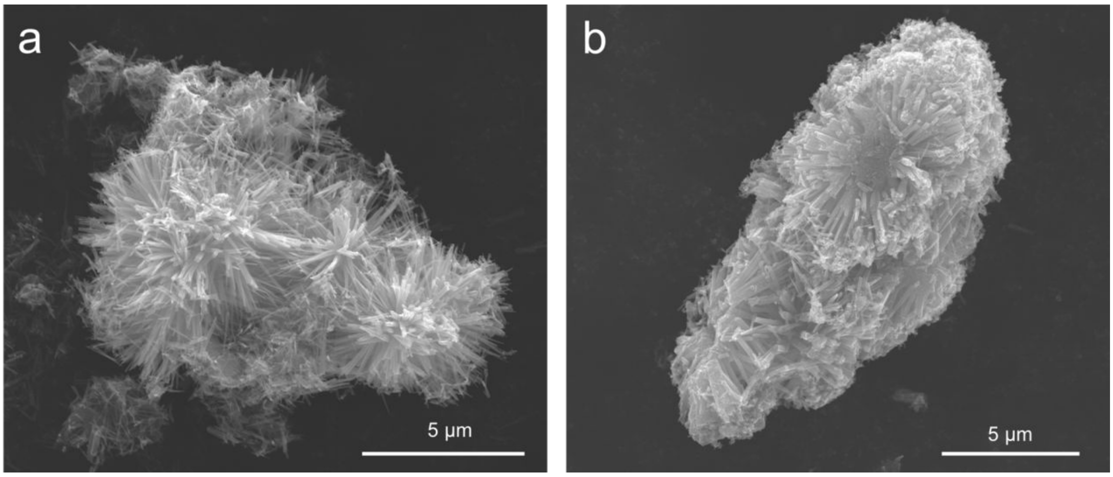

Figure S3. SEM images of $NiCo_2O_4$ at (a) pristine state and (b) after 10-hour UOR, showing similar "urchin-like" morphologies.

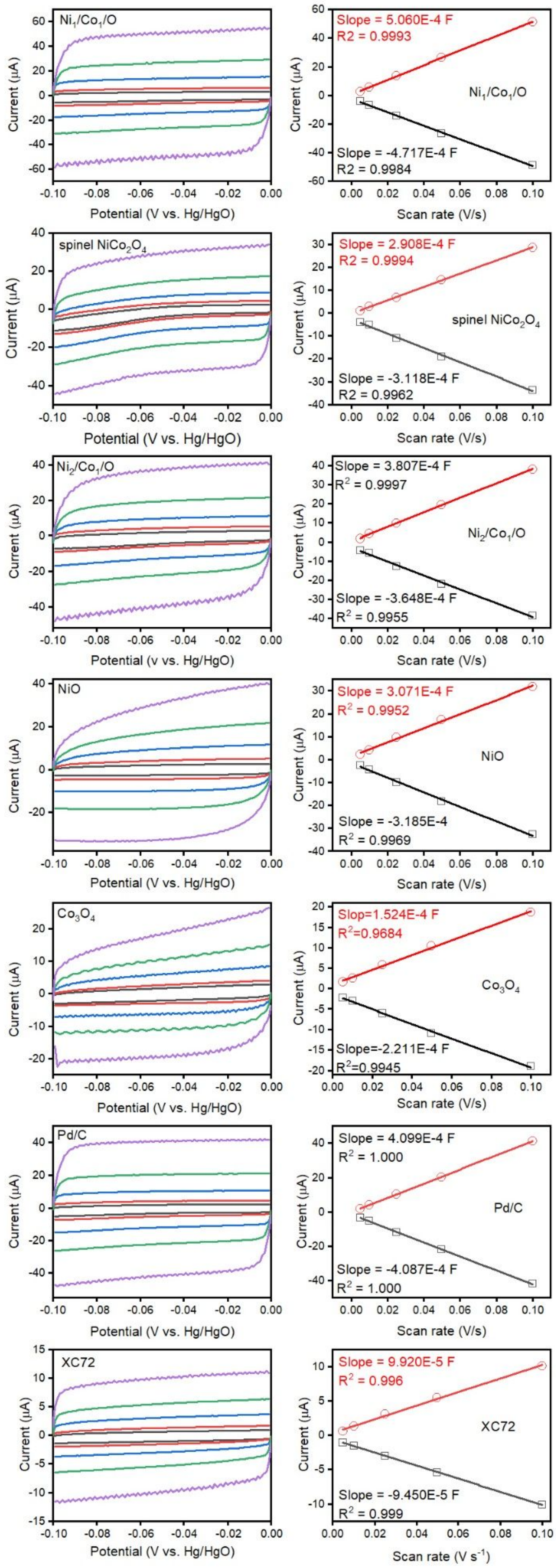

Figure S4. Double-layer capacitance measurements for different catalysts in 0.1M

NaOH at the following scan rates: (black line) 0.005 V/s, (red line) 0.01 V/s, (blue line) 0.025 V/s, (green line) 0.05 V/s, (purple line) 0.1 V/s. and their cathodic (black) and anodic (red) current measured at -0.05V vs Hg/HgO fitted linearly as a function of scan rate. The determined capacitance (C_{DL}) is averaged by the absolute value of the slopes and the final ECSA is calculated based on the equation: $ECSA(cm^2/g) = \frac{C_{DL}}{m \cdot C_s}$, where Cs is equal to 40 μF cm^{-2} and m is the loading amount (g).

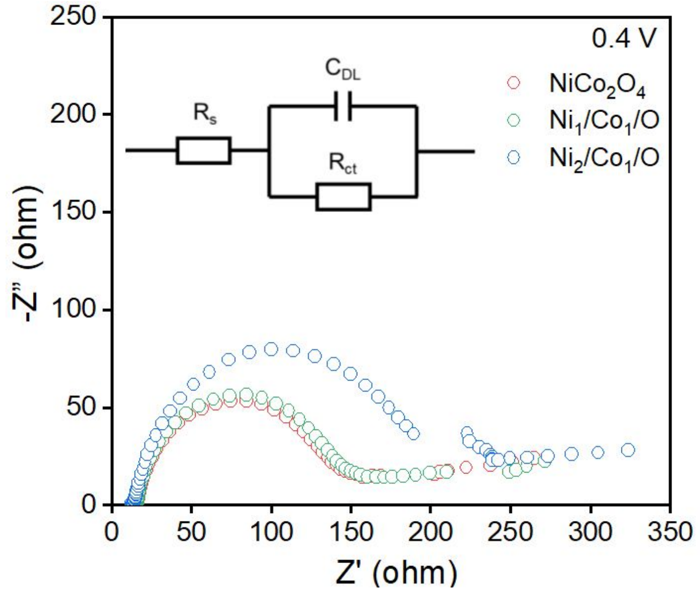

Figure S5: EIS measurements of pristine Ni/Co catalysts with various compositions.

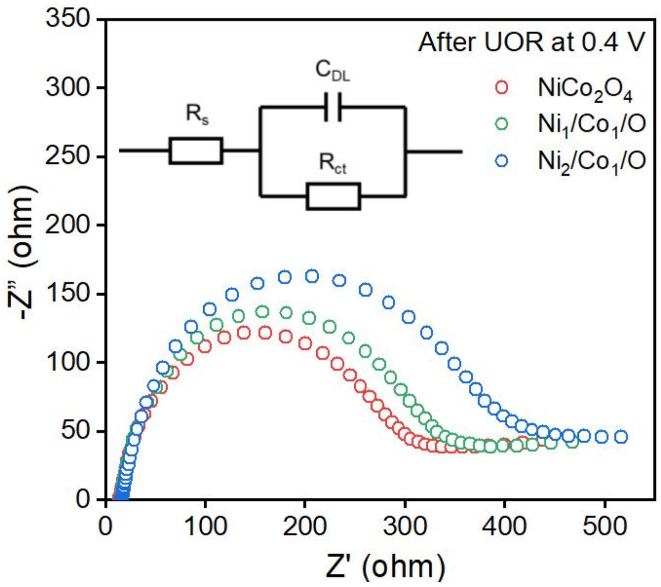

Figure S6: EIS measurements of Ni/Co catalysts with various compositions after UOR at 0.4 V.

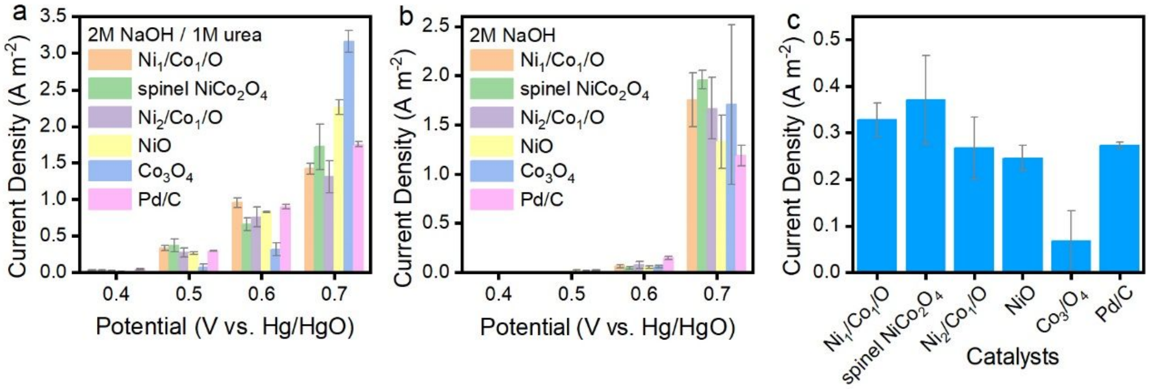

Figure S7. Anodic currents collected from the staircase voltammetry of different catalysts in (a) 2M NaOH/1M urea and (b) 2M NaOH, and (c) calculated UOR currents at 0.5V

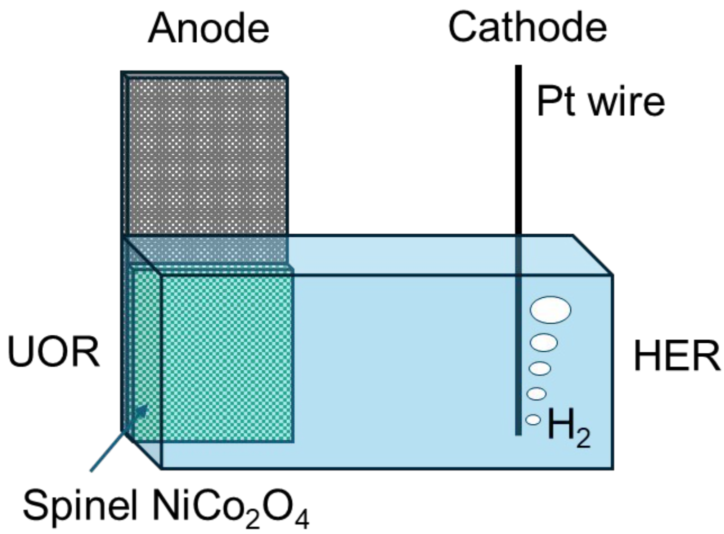

Figure S8. The scheme of the urea electrolysis cell setup.

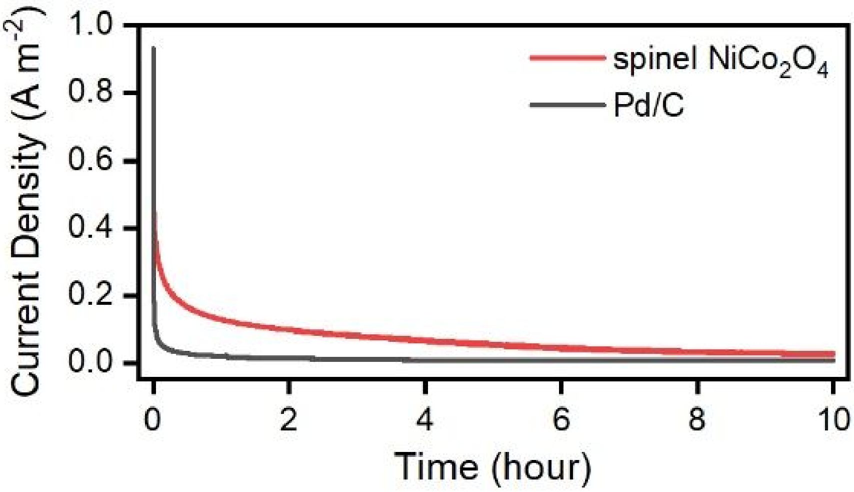

Figure S9. i-t long-term holding of NiCo2O4 and Pd at 0.5V in 2M NaOH/1M urea (GCE low loading).

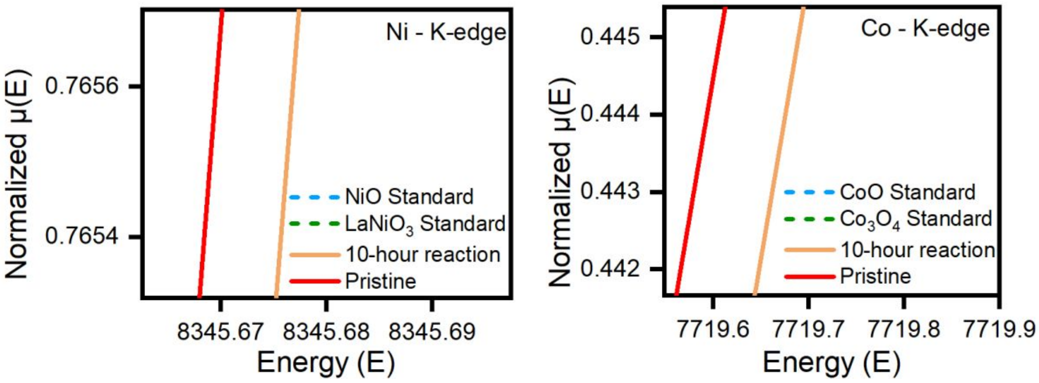

Figure S10. Zoomed-in XANES spectrum of Ni and Co K-edge.

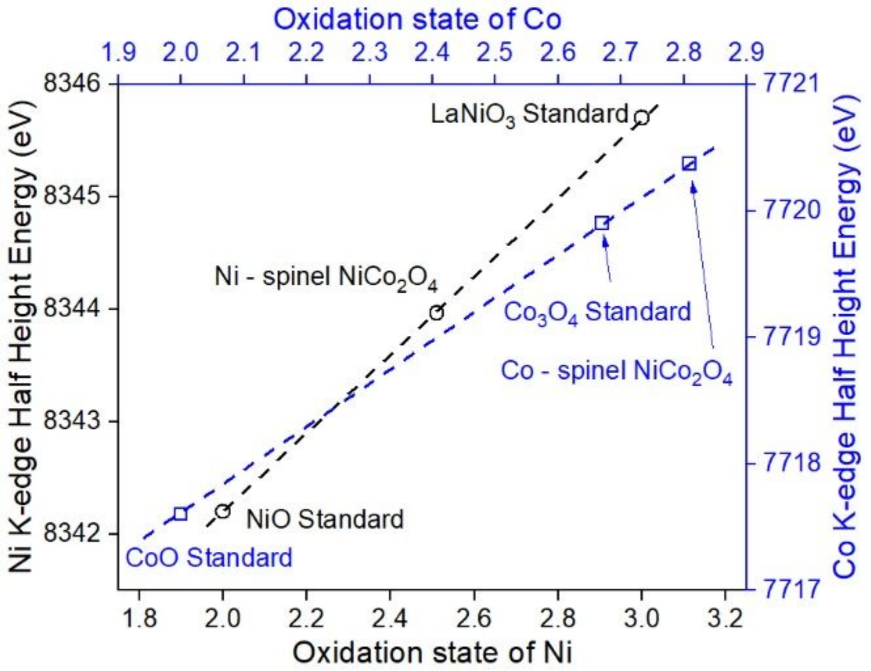

Figure S11. Valence calculation from the half-height energy value of Figure 5b,c.

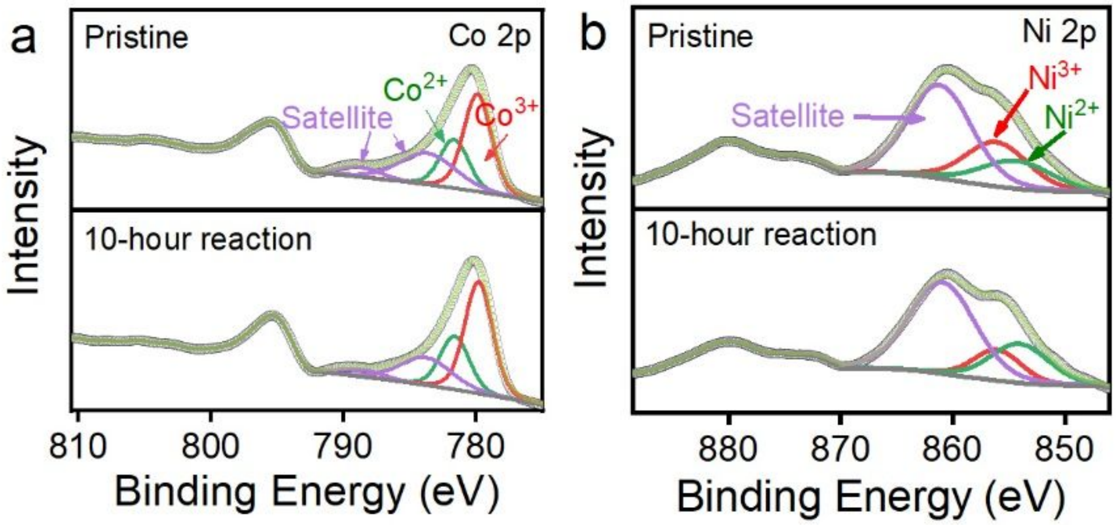

Figure S12. Full range XPS spectra (a) Co-2p and (b) Ni-2p components in spinel $NiCo_2O_4$ catalyst before and after long-term UOR.

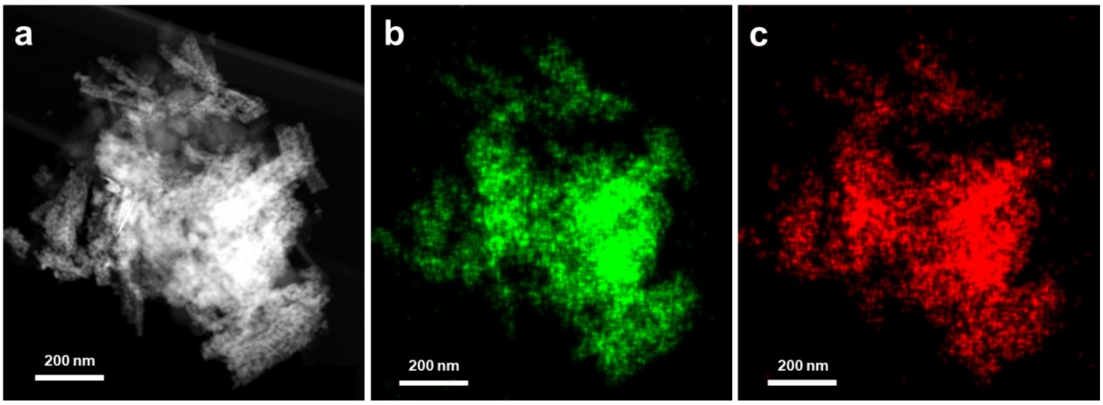

Figure S13. (a) HAADF image of the $NiCo_2O_4$ after 10-hour reaction with the element mapping of (b) Co and (c) Ni.

Table S1. Calculated ECSAs from Figure ~~S2~~S4.

**Catalyst**	$NiCo_2O_4$	Ni_1/Co_1-O	Ni_2/Co_1-O	NiO	Co_3O_4	Pd/C	XC72
**ECSA (m^2/g)**	36.5	70.0	49.3	38.6	16.0	94.4	40.3
